# The New Era in Dentistry

**Published:** 1904-12-15

**Authors:** C. M. Wright

**Affiliations:** Cincinnati, Ohio


### THE NEW ERA IN DENTISTRY.

BY DR. C. M. WRIGHT, CINCINNATI, OHIO.

The new era in dentistry, beginning with this century, presents three distinct fields for special cultivation in Prophylaxis, Orthodontia and Esthetic.

For the past third or quarter of a century the finely-finished gold "contour" filling has been held so near to the eyes of the dental profession that it has obscured the broad view of true medico-dental principles.

To be sure, there has been steady advancement in etiology, pathology and surgical therapeutics—also in the study of mal-occlusions of the teeth—and orthodontia, as well as in esthetics.

But these departments have been studied by a handful of men and have not become the universal basal ideas upon which the entire superstructure of dental practice must be built in the new era.

The American Dental Society of Europe stands among the first of the advance guard of progress. The program for this meeting, which has just reached me, affords evidence of this. The history of the work of individual members of this society shows alertness in prophetic perceptions of the "coming theories" of dental art and science.

The program shows that orthodontia, mal-occlusion and the esthetic porcelain art will occupy a large share of attention through papers and discussions. Last year, essays on preventive dentistry or prophylaxis were offered.

After forty years of continuous practice and observation of methods—and of fads and fashions—of dental practice, it seems clear to me that the new era will present these three as the most conspicuous and important principles upon which all our ideas of usefulness and success must depend. I should lay down the order as, First: special methods of prevention, such as *frequent and regular* skilled attention to the teeth, a la D. D. Smith. This is to consist of manipulative and surgical operations and medicinal and chemical applications by the dentist himself in anticipation of disease.

A proverb says, "Only the sick need a physician," but an aggressive conservatism now proclaims that active measures shall be employed *before* sickness develops. The sickness must be prevented. This is especially logical and legitimate in dental practice, for there is no department in sanitary medical practice in which the stitch in time can be more readily proved to save nine as in dental prophylaxis.

Second: In the same line of thought, orthodontia must occupy the attention of all practitioners instead of specialists in this department, as at present—to prevent evils which result from mal-occlusions and irregularities and also for esthetic reasons.

Third: The concealment of dental repairs by the use of porcelain instead of the glaring gold of the past fifteen or twenty years. The porcelain art era is definitely here.

These three fields, Preventive Dentistry, Orthodontia and the Porcelain Substitute, will require different methods of practice, different methods of teaching in dental schools and different methods of preaching at our chairs to patients; but our past studies and acquired skill in other directions as a profession will widen the gates and progress will result according to the natural law of evolution.—*Review.*

